# Supplementary material for: Psychometric Testing of the CEECCA Questionnaire to Assess Ability to Communicate among Individuals with Aphasia
Source: Int J Environ Res Public Health. 2023 Feb 22;20(5):3935. doi: 10.3390/ijerph20053935 (PMC10001674; doi:10.3390/ijerph20053935)
Supplement: Supplementary file 1 [file ijerph-20-03935-s001.zip › ijerph-2181860-supplementary.pdf]

CUESTIONARIO PARA LA EVALUACIÓN ENFERMERA DE LA CAPACIDAD COMUNICATIVA EN LA AFASIA

CEECCA

CEECCA

CUESTIONARIO PARA LA EVALUACIÓN ENFERMERA DE LA CAPACIDAD COMUNICATIVA EN LA AFASIA

ANAMNESIS

Fecha de evaluación: ______________________

Lugar de evaluación (marcar con una X):

Hospital ☐ Centro de Salud ☐ Domicilio ☐ Otro ☐

Edad: __________

Sexo (marcar con una X):

Varón ☐ Mujer ☐

Estado Civil (marcar con una X):

Soltero ☐ Casado ☐ Viudo/a ☐

Nivel de estudios (marcar con una X):

Nulo ☐ Medio ☐ Bueno ☐ Muy bueno ☐

Nivel de Escritura previo (marcar con una X):

Nulo ☐ Medio ☐ Bueno ☐ Muy bueno ☐

Antecedentes ocupacionales y profesión: ______________________

____

Idioma (marcar con una X):

Sólo Español ☐ Biligüe/Multilingüe ☐ Idiomas: __________

Vive solo (marcar con una X):

Si ☐ No ☐ Soporte Familiar: Si ☐ No ☐

Antecedentes Personales:

____

____

____

____

Presencia de Hipoacusia (marcar con una X):

Si ☐ No ☐ Corregida: Si ☐ No ☐

Presencia de alteraciones de la visión (marcar con una X):

Si ☐ No ☐ Corregida: Si ☐ No ☐

Etiología de la Afasia (marcar con una X):

ACV Isquémico ☐ ACV Hemorrágico ☐ TCE ☐ Tumor ☐

Infección SNC ☐ Intervención Quirúrgica ☐

Enf. Neurodegenerativa ☐ Otra ☐

Fecha de la lesión: ____________________

Localización de la lesión: ______________________________________

Tipo de afasia: ____

Recibe Rehabilitación (marcar con una X):

Si ☐ Tipo: ____

Tiempo: _____________________ No ☐

Ha recibido Rehabilitación (marcar con una X):

Si ☐ Tipo: ____

Tiempo: _____________________ Fecha finalización: ____________________ No ☐

Mano dominante (marcar con una X):

Derecha ☐ Izquierda ☐

Presencia de Hemiplejía ☐

(puede marcar con una X más de una opción):

Derecha ☐ Izquierda ☐ Recuperada ☐ Ausente ☐

Presencia de Hemiparesia ☐

(puede marcar con una X más de una opción):

Derecha ☐ Izquierda ☐ Recuperada ☐ Ausente ☐

Presencia de Hemianopsia ☐

(puede marcar con una X más de una opción):

Derecha ☐ Izquierda ☐ Recuperada ☐ Ausente ☐

1. 1. Habla en conversación.

Instrucción: Procure un ambiente que propicie la conversación. Inicie la misma incorporando las preguntas que se recogen en el apartado "Habla en conversación" del cuestionario. Siempre que sea posible, cuando se formulen preguntas abiertas se debe estimular al paciente para inducir secuencias de lenguaje lo más elaboradas posibles. Pase a la siguiente pregunta cuando considere que tiene información para registrar el nivel de ejecución de acuerdo con los cuatro niveles propuestos en la hoja de respuesta.

Ítem 1. Respuesta abierta. Pregunte: "¿Cómo se encuentra usted hoy?"

Ítem 2. Pregunte: "¿Cuál es su nombre completo?"

Ítem 3. Pregunte (Elija una o similar): "¿Cuál es su dirección?" "¿Dónde vive usted?"

Ítem 4. Respuesta si/no. Pregunte (Elija una o similar): "¿Está usted muy cansado hoy?" "¿Ha descansado usted bien esta noche?"

Ítem 5. Respuesta abierta. Pídale (Elija una o similar): "Cuénteme a qué se dedica/dedicaba usted" "Explíqueme en qué consiste/consistía su trabajo"

Puntuación (ítems 1-5):
Para cada ítem, marque con una "X" en la hoja de registro el nivel de ejecución que corresponda de acuerdo a la respuesta dada.

1. 2. Habla de exposición.

Ítem 6. Descripción de la lámina *"La caída del pintor"*.

Instrucción: Muestre al paciente la **lámina 1** e indíquele: *"Dígame todo lo que vea que está sucediendo en esta lámina"*. Permita que el paciente se exprese como máximo *durante tres minutos*. Si cree que facilita la expresión, puede utilizar ayudas neutras del tipo: "¿Qué ve usted aquí?", "¿Ve algo más por aquí?". No use ayudas no neutras como: "¿Qué le ocurre al pintor?".

Para evaluar este ítem necesita cuantificar las unidades funcionales de contenido (UFC) correctas que produzca el paciente. Se entiende como *unidad funcional de contenido* aquellas unidades descritas o unidades nuevas de información proporcionadas por la persona que describan elementos (objetos, acciones, situaciones, ...) correctos de la lámina. Sólo debe contar la mejor producción de una unidad de contenido.

A continuación, se facilita una lista con 14 UFC principales, sin embargo, no es necesario que el paciente exprese la UFC tal y como aparece en la lista, pudiendo trasmitir la misma o similar información usando una estructura con una organización gramatical diferente.

Puede facilitar esta tarea marcar con una "X" en los recuadros inferiores las UFC correctas a medida que se vayan produciendo.

UNIDADES FUNCIONALES DE CONTENIDO	MARQUE CON UNA X
El pintor se cae de la escalera.	☐
El pintor cae sobre la carretilla.	☐
El pintor estaba pintando la casa.	☐
Al pintor se le cae la brocha.	☐
Al pintor se le cae la gorra.	☐
El pintor intenta coger la brocha.	☐
La brocha se cae.	☐
La gorra se cae.	☐
El cubo se cae.	☐
La pintura se derrama.	☐
El obrero golpea la escalera.	☐
El obrero tira al pintor.	☐
El perro corre detrás del gato.	☐
Hay un avión volando	☐

Puntuación (ítem 6):
Marque con una "X" en la hoja de registro el nivel de ejecución que corresponda de acuerdo con la respuesta dada.

LÁMINA 1

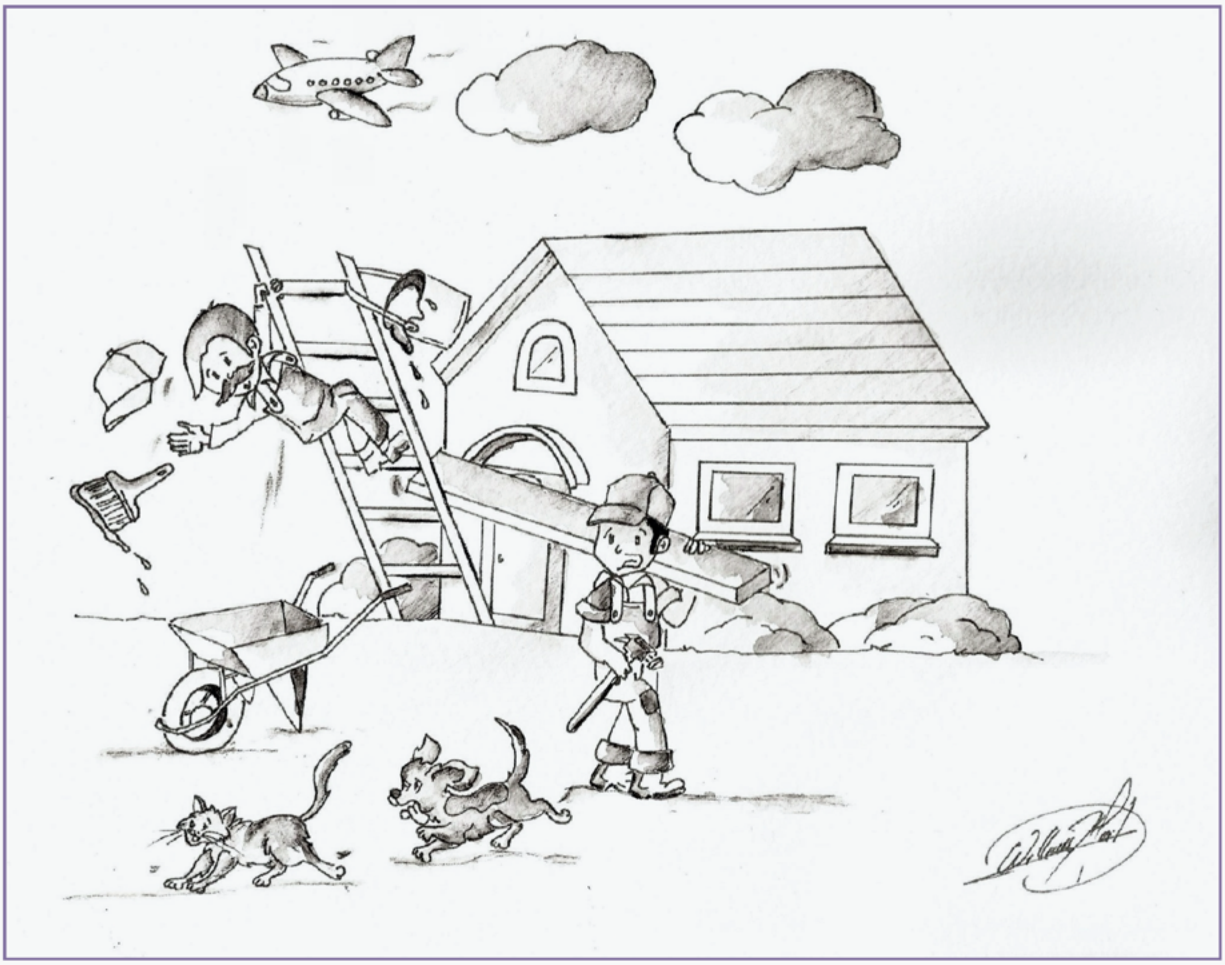

1. 3. Denominación oral.

Ítems 7 - 10. Denominación oral de objetos. **Lámina 2** (4 imágenes).

Ítems 11 -13. Denominación oral de acciones. **Lámina 3** (3 imágenes).

Instrucción: Coloque la lámina de manera que se asegure una buena visibilidad de la misma (Considerar posible hemianopsia). Para denominación de objetos (ítems 7 - 10), señale cada imagen e indique: "Dígame, qué es esto". Para denominación de acciones (ítems 11 -13), señale cada imagen e indique: "Dígame, qué se está haciendo en esta imagen". Si el paciente lo pide, se puede repetir la indicación una vez más. Permita como máximo 10 segundos para elaborar cada respuesta, pasado ese tiempo la respuesta se considera disfuncional. Siga el orden indicado.

Puntuación (Ítems 7-13):
Para cada ítem, marque con una "X" en la hoja de registro el nivel de ejecución que corresponda de acuerdo con la respuesta dada.

LÁMINA 2

Ítem 7

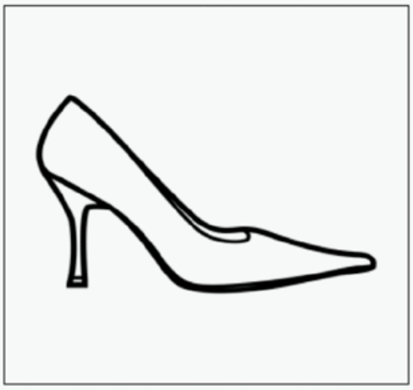

Ítem 8

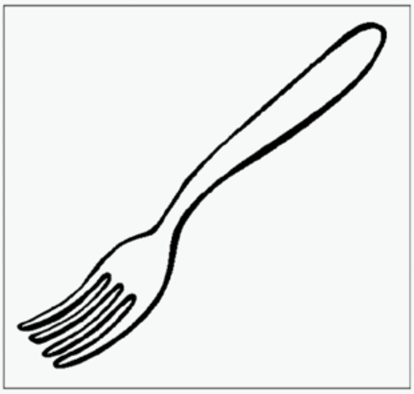

Ítem 9

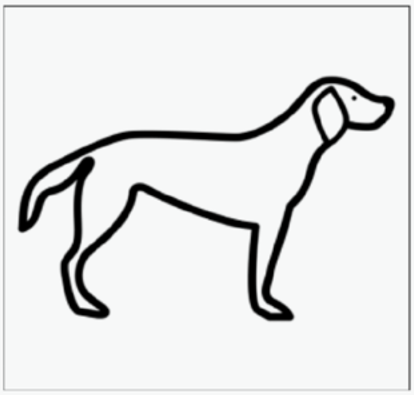

Ítem 10

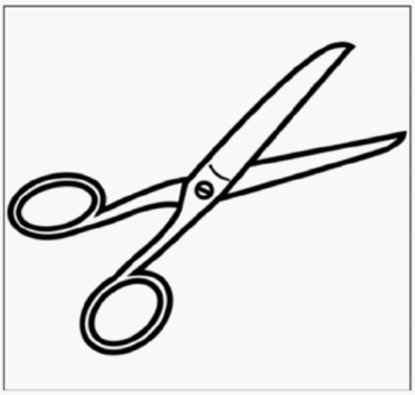

LÁMINA 3

Ítem 11

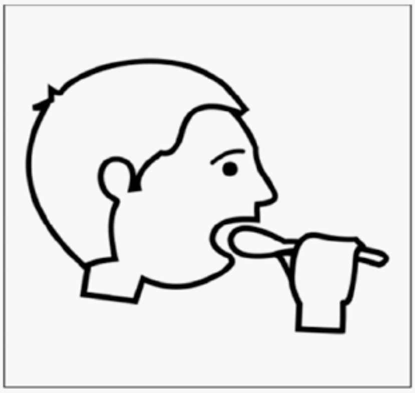

Ítem 12

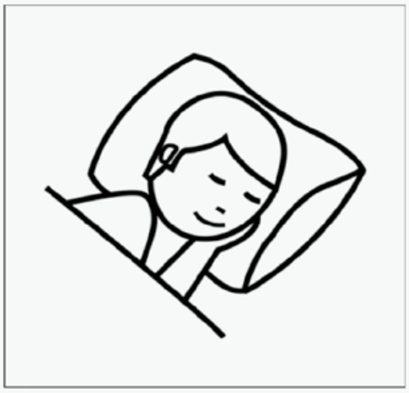

Ítem 13

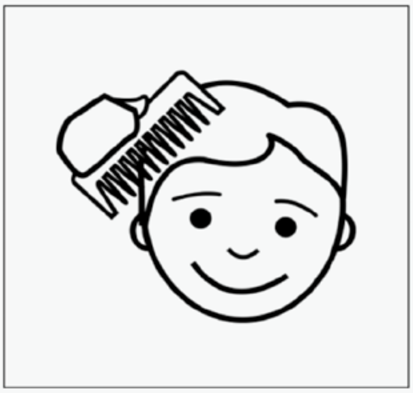

2. 1. Escritura del nombre y apellidos.

Ítem 14. Pídale que escriba su nombre y apellidos completo.

Instrucción: Proporcione un lápiz o bolígrafo y papel. Si existe déficit motor, asegúrese que puede coger el lápiz o el bolígrafo con la mano que tenga mejor movilidad. Situar al paciente de manera cómoda para facilitar la tarea de escritura. Coloque la lámina de manera que se consiga buena visibilidad de la misma (Considerar posible hemianopsia). Una vez se inicie la escritura permita un máximo de 1 minuto para elaborar la respuesta, pasado ese tiempo la respuesta se considera disfuncional.

Puntuación (ítems 14):
Marque con una "X" en la hoja de registro el nivel de ejecución que corresponda de acuerdo con la respuesta dada.

2. 2. Denominación escrita.

Ítems 15 - 17. Denominación escrita de objetos. **Lámina 4** (3 imágenes).

Ítems 18 - 20. Denominación escrita de acciones. **Lámina 5** (3 imágenes).

Instrucción: Proporcione un lápiz o bolígrafo y papel. Si existe déficit motor, asegúrese que puede coger el lápiz o el bolígrafo con la mano que tenga mejor movilidad. Situar al paciente de manera cómoda para facilitar la tarea de escritura. Coloque la lámina de manera que se consiga buena visibilidad de la misma (considerar posible hemianopsia). Para denominación escrita de objetos (ítems 15-17), señale cada imagen e indique: "Escriba el nombre de esto". Para denominación escrita de acciones (ítems 18-20), señale cada imagen e indique: "Escriba lo que está haciendo esta persona". Si el paciente lo pide, se puede repetir la indicación una vez más. Permita como máximo 30 segundos para elaborar cada respuesta, pasado ese tiempo la respuesta se considera disfuncional. Siga el orden indicado.

Puntuación (Ítems 15 - 20):
Para cada ítem, marque con una "X" en la hoja de registro el nivel de ejecución que corresponda de acuerdo con la respuesta dada.

LÁMINA 4

Ítem 15

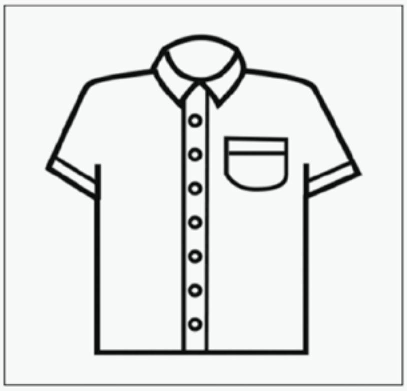

Ítem 16

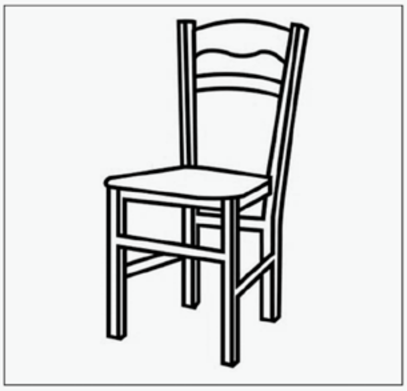

Ítem 17

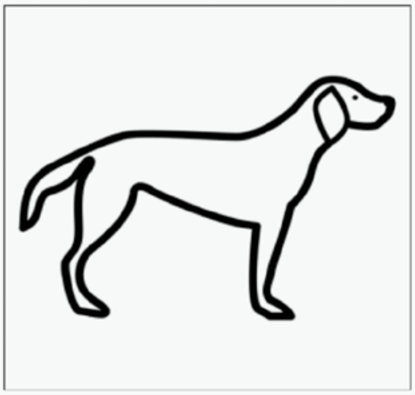

LÁMINA 5

Ítem 18

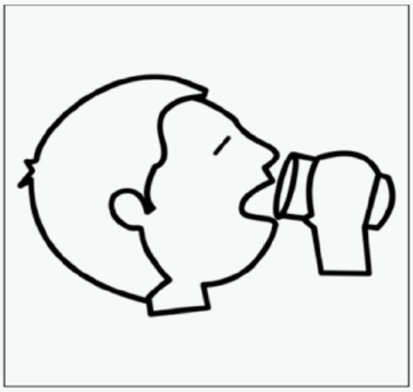

Ítem 19

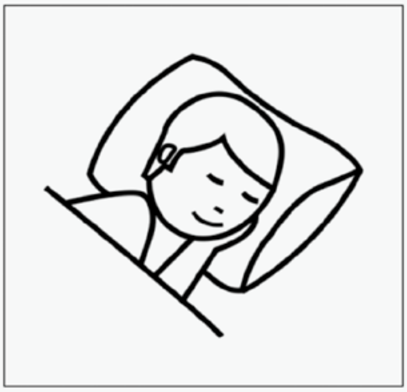

Ítem 20

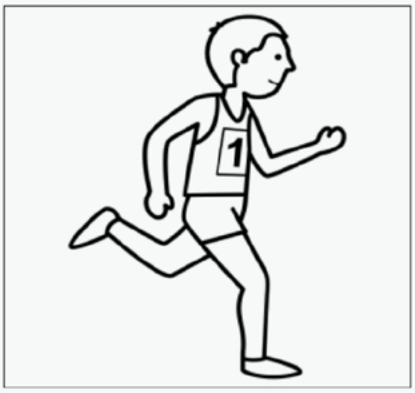

3. EXPRESIÓN A TRAVÉS DE PICTOGRAMAS.

Ítems 21 - 23. Expresión de acciones a través de pictogramas. **Lámina 6** (3 imágenes).

Instrucción: Asegure la posición cómoda para del paciente. Coloque la lámina de manera que consiga buena visibilidad de la misma (Considerar posible hemianopsia). Coloque la lámina al alcance de la mano útil del paciente considerando posible dificultad por déficit motor. *Permita como máximo 10 segundos* para elaborar cada respuesta, pasado ese tiempo la respuesta se considera disfuncional.

Indique: " Si usted .... ¿Qué dibujo elegiría?". Si el paciente lo pide, se puede repetir la indicación una vez más. Siga el orden indicado:

1) Ítem 21. Quisiera COMER.
2) Ítem 22. Quisiera DUCHARSE.
3) Ítem 23. Quisiera LEER.

Ítems 24 - 26. Expresión de emociones a través de pictogramas. **Lámina 7** (3 imágenes).

Instrucción: Indique: " Si usted .... ¿Qué dibujo elegiría?". Si el paciente lo pide, se puede repetir la indicación una vez más. *Permita como máximo 10 segundos* para elaborar cada respuesta, pasado ese tiempo la respuesta se considera disfuncional.

Siga el orden indicado:

1) Ítem 24. Estuviese ALEGRE.
2) Ítem 25. Estuviese TRISTE.
3) Ítem 26. Estuviese ENFADADO

Puntuación (Ítems 21 - 26):
Para cada ítem, marque con una "X" en la hoja de registro el nivel de ejecución que corresponda de acuerdo a la respuesta dada.

Ítem 23

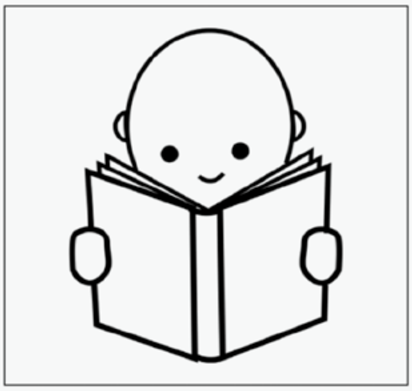

Ítem 21

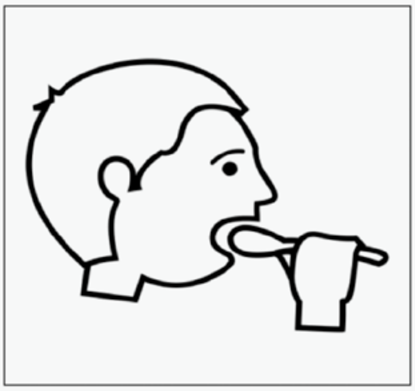

Ítem 22

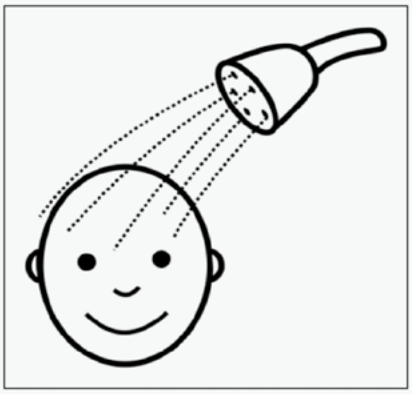

EXPRESIÓN

LÁMINA 7

Ítem 26

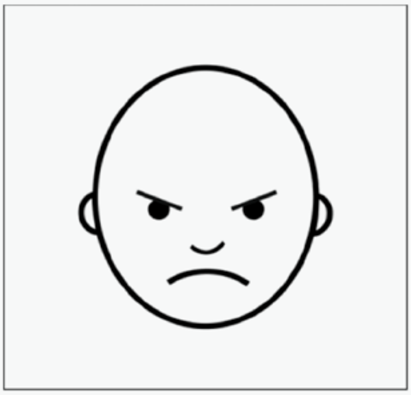

Ítem 24

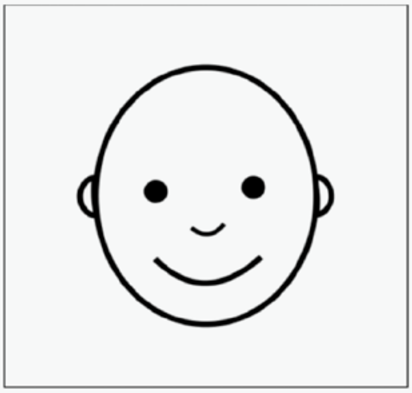

Ítem 25

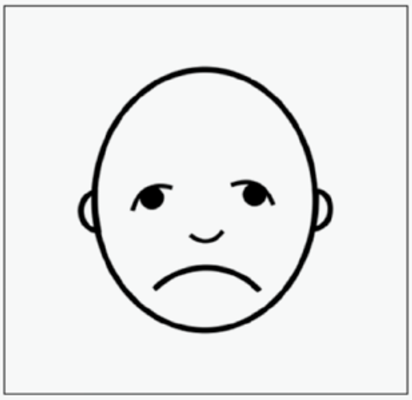

1. COMPRENSIÓN AUDITIVA

1. 1. Comprensión auditiva de palabras

Ítems 27 - 31. Reconocimiento auditivo de palabras. **Lámina 8** (5 imágenes).

Instrucción: Indique: "Yo le voy a decir el nombre de un objeto y usted lo señala". Si el paciente lo pide o si el examinador cree que puede ser útil, se puede repetir la palabra una vez más. Permita como máximo 10 segundos para elaborar cada respuesta, pasado ese tiempo la respuesta se considera disfuncional. Siga el orden indicado:

1) **Ítem 27.** Peine
2) **Ítem 28.** Tenedor
3) **Ítem 29.** Pera
4) **Ítem 30.** Mano
5) **Ítem 31.** Pantalón

Puntuación (Ítems 27 - 31):
Para cada ítem, marque con una "X" en la hoja de registro el nivel de ejecución que corresponda de acuerdo a la respuesta dada.

LÁMINA 8

Ítem 28

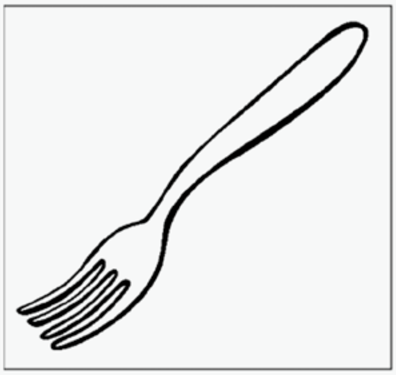

Ítem 29

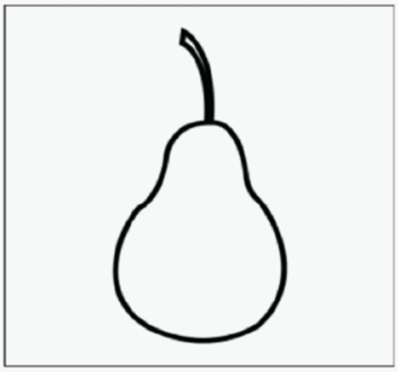

Ítem 27

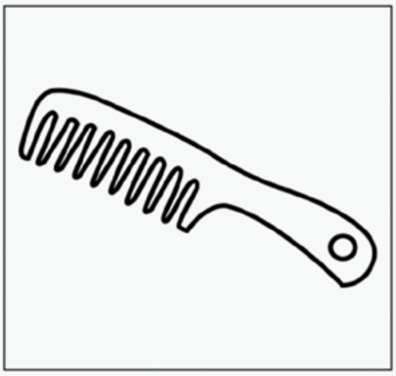

Ítem 31

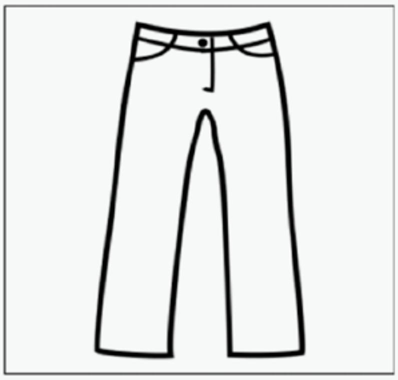

Ítem 30

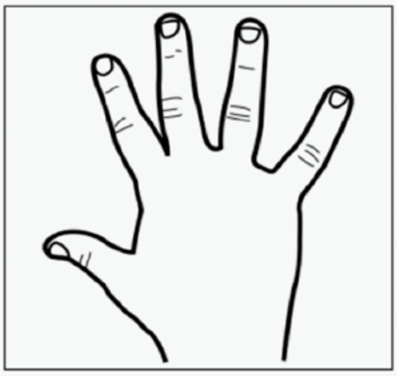

1. 2. Comprensión auditiva de oraciones.

Ítems 32 - 34. Lámina 9, 10 y 11.

Instrucción: Indique: *"Yo le voy a decir una frase y le voy a enseñar tres imágenes. Usted me tiene que decir a qué imagen se corresponde la frase".* Cada oración debe expresarse de forma completa, sin dividir y con lenguaje claro. Si el paciente lo pide o si el examinador cree que puede ser útil, se puede repetir la oración una vez más. *Permita como máximo 10 segundos* para elaborar cada respuesta, pasado ese tiempo la respuesta se considera disfuncional.

Cada lámina contiene tres imágenes. Cada imagen se describe con una oración. El examinador debe elegir una de las tres oraciones por lámina y expresarla oralmente al paciente para que éste señale la imagen que corresponda.

Ítem 32 (Lámina 9):

- El banco está entre tres farolas.
- La farola está entre tres bancos.
- El banco está entre dos farolas.

Ítem 33 (Lámina 10):

- La pelota está debajo de la mesa que está entre la silla y el armario.
- La pelota está sobre la silla que está entre la mesa y el armario.
- La pelota está sobre la mesa que está entre el armario y la silla.

Ítem 34 (Lámina 11):

- El coche grande circula detrás de la bicicleta y delante del coche pequeño.
- El coche grande circula detrás del coche pequeño y delante de la bicicleta.
- El coche pequeño circula detrás de la bicicleta y delante del coche grande.

Puntuación (Ítems 32 - 34):
Para cada ítem, marque con una "X" en la hoja de registro el nivel de ejecución que corresponda de acuerdo a la respuesta dada.

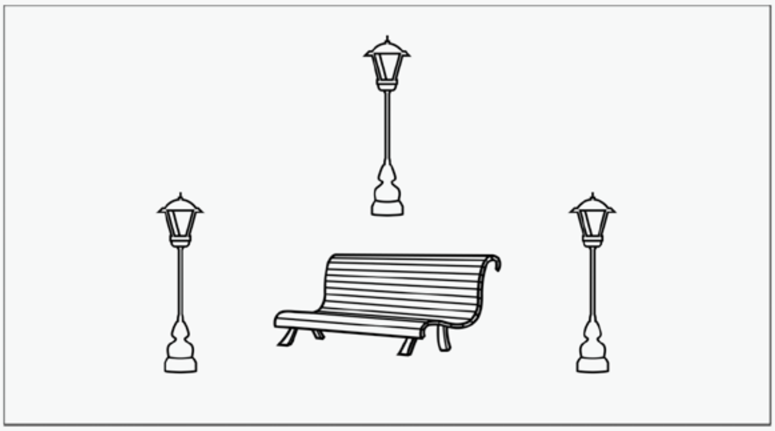

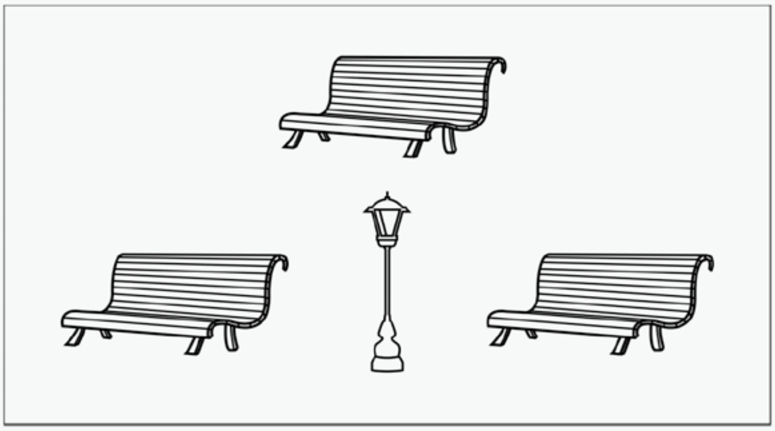

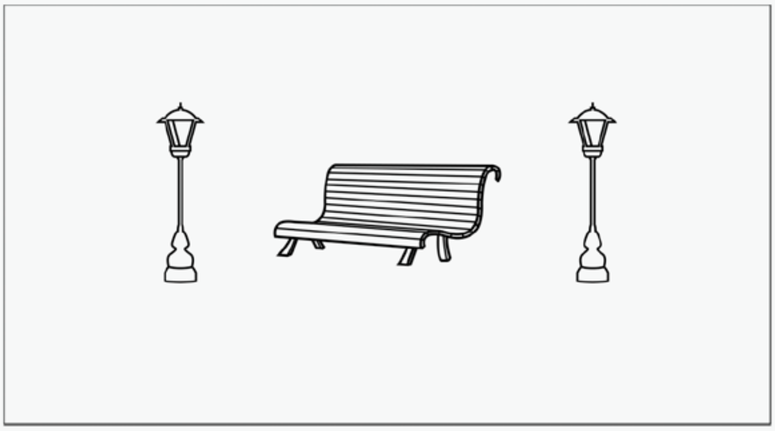

Ítem 32

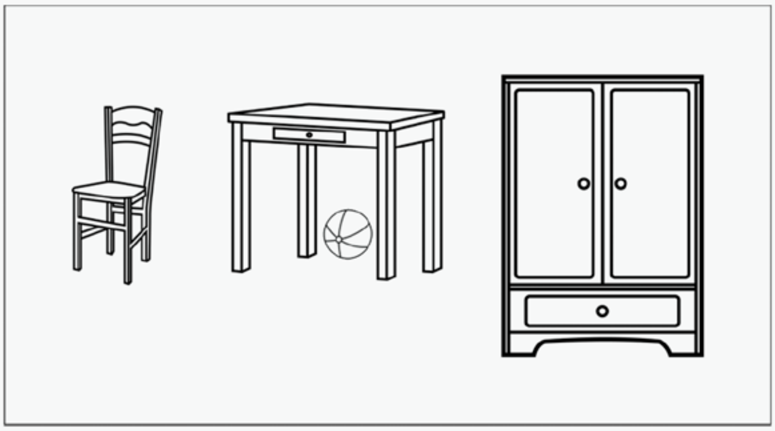

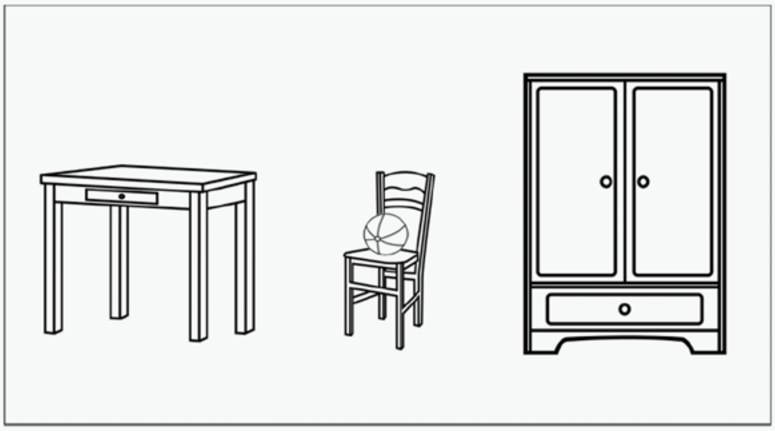

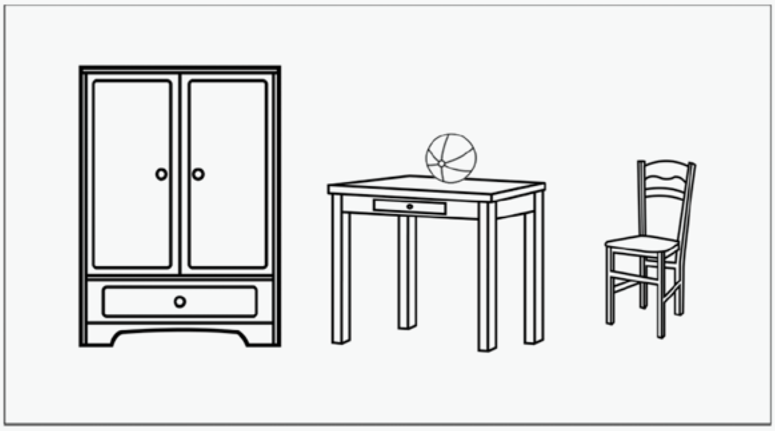

Ítem 33

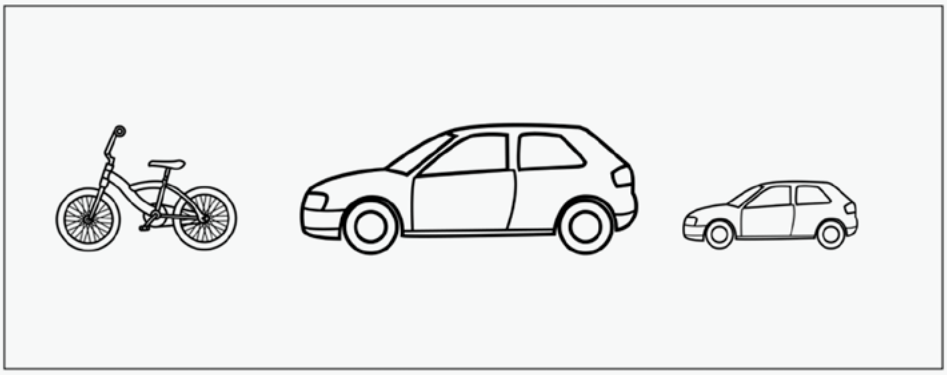

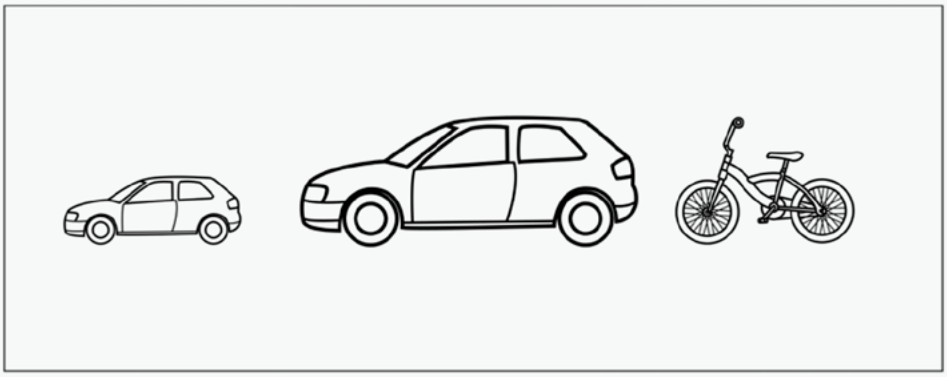

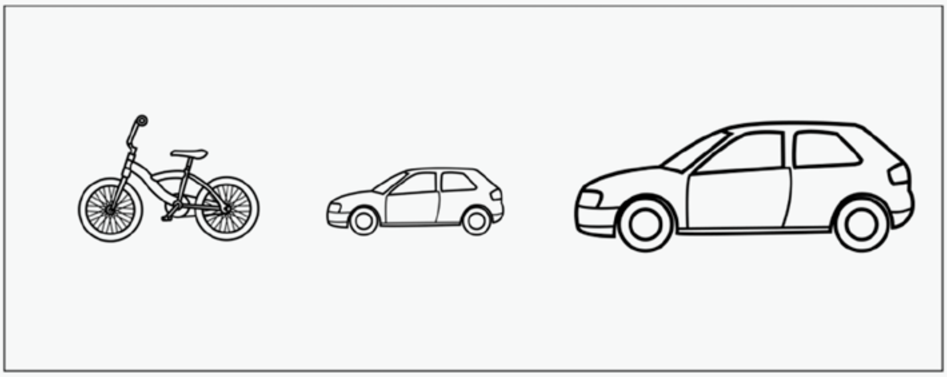

Ítem 34

1. 3. Comprensión auditiva de órdenes.

Ítems 35 - 37. Comprensión de órdenes verbales:

Instrucción: Indique: *"Siga las instrucciones que yo le diga"*. Las órdenes deben expresarse de forma completa, sin dividir. Si el paciente lo pide o el examinar cree que puede ser útil, se puede repetir la frase una vez más, siempre como un todo. *Permita como máximo 10 segundos* para elaborar cada respuesta, pasado ese tiempo la respuesta se considera disfuncional. Siga el orden indicado:

1) Ítem 35. Mire al techo
2) Ítem 36. Levante la mano y luego señale el lápiz (o bolígrafo)
3) Ítem 37. Tóquese la oreja, luego tóquese la nariz y luego cierre los ojos.

Puntuación (Ítems 35 - 37):
Para cada ítem, marque con una "X" en la hoja de registro el nivel de ejecución que corresponda de acuerdo a la respuesta dada.

2.1. Comprensión lectora de palabras

Ítems 38 - 40. Relación imagen - palabra escrita. **Lámina 12 y 13.**

Instrucción: Indique: *"Ahora le voy a enseñar un dibujo y cuatro palabras. Dígame qué palabra corresponde al dibujo". Permita como máximo 15 segundos* para elaborar cada respuesta, pasado ese tiempo la respuesta se considera disfuncional. Siga el orden indicado.

Puntuación (ítems 38 - 40):
Para cada ítem, marque con una "X" en la hoja de registro el nivel de ejecución que corresponda de acuerdo a la respuesta dada.

2.2. Comprensión lectora de oraciones

Ítems 41 - 43. Comprensión de oraciones escritas. **Lámina 14.**

Instrucción: Indique: *"Ahora va a leer una frase que está sin acabar. Señale con el dedo la palabra adecuada para completarla". Permita como máximo 15 segundos* para elaborar cada respuesta, pasado ese tiempo la respuesta se considera disfuncional. Siga el orden indicado.

Puntuación (Ítems 41 - 43):
Para cada ítem, marque con una "X" en la hoja de registro el nivel de ejecución que corresponda de acuerdo a la respuesta dada.

Ítem 38

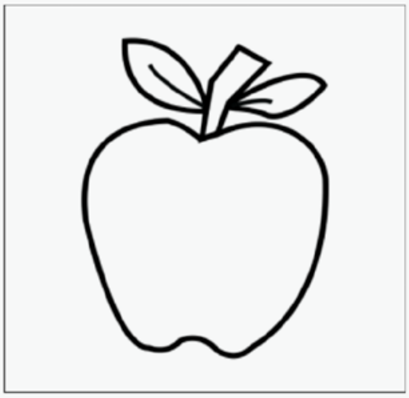

MARACA	PERA	MANZANA	CUCHARA

Ítem 39

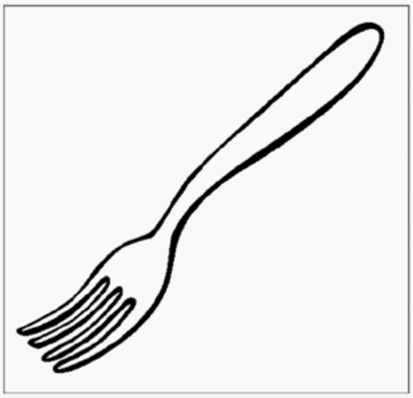

CUCHARA	TENEDOR	SOPA	TELEVISOR

Ítem 40

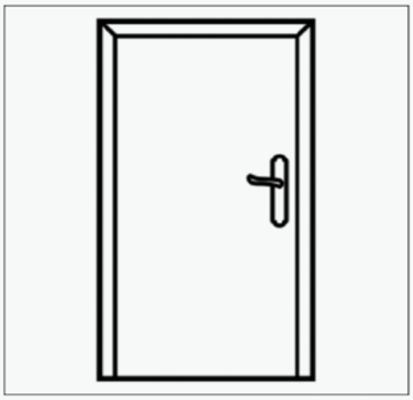

PUERTA	PUENTE	TEJADO	VENTANA

Ítem 41

LA SILLA TIENE CUATRO...

MADERA	PATAS	COCINA	COMEDOR

Ítem 42

PARA HACER UNA TORTILLA NECESITO...

SOL	RESTAURANTE	HUEVOS	GRANJA

Ítem 43

JUAN ES MÚSICO, PASA MUCHAS HORAS CANTANDO Y TOCANDO LA...

GUITARRA	DISCOTECA	MICRÓFONO	BRAZO

HOJAS DE RESULTADOS

CEECCA

EXPRESIÓN

EXPRESIÓN ORAL

HABLA EN CONVERSACIÓN	DISFUNCIONAL		FUNCIONAL	
	Ausencia de habla.	Respuesta incomprensible o inadecuada. No es posible la inferencia.	Respuesta parcialmente comprensible y adecuada. Es posible la inferencia.	Respuesta comprensible y adecuada.
Ítem 1	☐	☐	☐	☐
Ítem 2	☐	☐	☐	☐
Ítem 3	☐	☐	☐	☐
Ítem 4	☐	☐	☐	☐
Ítem 5	☐	☐	☐	☐
**Recuento Respuestas**				
**Etiqueta diagnóstica: Habla en conversación disfuncional**	Si dos o más respuestas son disfuncionales			

HABLA EN EXPOSICIÓN	DISFUNCIONAL		FUNCIONAL	
	Ausencia de habla.	Exposición incomprensible o que no describe la imagen con al menos cuatro unidades de contenido adecuadas. No es posible la inferencia.	Exposición parcialmente comprensible. Describe la imagen con al menos cuatro unidades de contenido. Es posible la inferencia.	Exposición comprensible. Describe la imagen con cuatro o más unidades de contenido comprensibles
Ítem 6	☐	☐	☐	☐
**Recuento Respuestas**				
**Etiqueta diagnóstica: Habla en exposición disfuncional**	Si la respuesta es disfuncional			

DENOMINACIÓN ORAL DE OBJETOS	DISFUNCIONAL		FUNCIONAL	
	Ausencia de habla.	Respuesta incomprensible o inadecuada. No posible la inferencia. Respuesta en > 10´´	Respuesta parcialmente comprensible y adecuada. Es posible la inferencia.	Respuesta comprensible y adecuada.
**Ítem 7**	☐	☐	☐	☐
**Ítem 8**	☐	☐	☐	☐
**Ítem 9**	☐	☐	☐	☐
**Ítem 10**	☐	☐	☐	☐
**Recuento Respuestas**				
**Etiqueta diagnóstica: Denominación oral de objetos disfuncional**	Si dos o más respuestas son disfuncionales			

DENOMINACIÓN ORAL DE ACCIONES	DISFUNCIONAL		FUNCIONAL	
**Ítem 11**	☐	☐	☐	☐
**Ítem 12**	☐	☐	☐	☐
**Ítem 13**	☐	☐	☐	☐
**Recuento Respuestas**				
**Etiqueta diagnóstica: Denominación oral de acciones disfuncional**	Si dos o más respuestas son disfuncionales			

EXPRESIÓN ESCRITA

ESCRITURA DEL NOMBRE	DISFUNCIONAL		FUNCIONAL	
	Ausencia de escritura.	Respuesta no legible o inadecuada. No posible la inferencia. Respuesta en > 1min	Respuesta parcialmente legible y adecuada. Es posible la inferencia.	Respuesta completa y adecuada
**Ítem 14**				
**Recuento Respuestas**				
**Etiqueta diagnóstica: Escritura del nombre disfuncional**	Si la respuesta es disfuncional			

DENOMINACIÓN ESCRITA DE OBJETOS	DISFUNCIONAL		FUNCIONAL	
	Ausencia de escritura.	Respuesta no legible o inadecuada. No posible la inferencia. Respuesta en > 30''	Respuesta parcialmente legible y adecuada. Es posible la inferencia.	Respuesta completa y adecuada
**Ítem 15**				
**Ítem 16**				
**Ítem 17**				
**Recuento Respuestas**				
**Etiqueta diagnóstica: Denominación escrita de objetos disfuncional**	Si dos o más respuestas son disfuncionales			

DENOMINACIÓN ESCRITA DE ACCIONES	DISFUNCIONAL		FUNCIONAL	
**Ítem 18**				
**Ítem 19**				
**Ítem 20**				
**Recuento Respuestas**				
**Etiqueta diagnóstica: Denominación escrita de acciones disfuncional**	Si dos o más respuestas son disfuncionales			

EXPRESIÓN DE ACCIONES A TRAVÉS DE PICTOGRAMAS	DISFUNCIONAL		FUNCIONAL
	Ausencia de respuesta.	Señala imagen incorrecta. Respuesta en > 10´´	Señala la imagen correcta.
**Ítem 21**	☐	☐	☐
**Ítem 22**	☐	☐	☐
**Ítem 23**	☐	☐	☐
**Recuento Respuestas**			
**Etiqueta diagnóstica: Expresión de acciones a través de pictogramas disfuncional**	Si dos o más respuestas son disfuncionales		

EXPRESIÓN DE EMOCIONES A TRAVÉS DE PICTOGRAMAS	DISFUNCIONAL		FUNCIONAL
**Ítem 24**	☐	☐	☐
**Ítem 25**	☐	☐	☐
**Ítem 26**	☐	☐	☐
**Recuento Respuestas**			
**Etiqueta diagnóstica: Expresión de emociones a través de pictogramas disfuncional**	Si dos o más respuestas son disfuncionales		

COMPRENSIÓN

COMPRENSIÓN AUDITIVA

COMPRENSIÓN AUDITIVA DE PALABRAS	DISFUNCIONAL		FUNCIONAL
	Ausencia de respuesta o nula comprensión auditiva.	Señala imagen incorrecta. Respuesta en > 10˝	Señala la imagen correcta.
**Ítem 27**			
**Ítem 28**			
**Ítem 29**			
**Ítem 30**			
**Ítem 31**			
**Recuento Respuestas**			
**Etiqueta diagnóstica: Comprensión auditiva de palabras disfuncional**	Si dos o más respuestas son disfuncionales		

COMPRENSIÓN AUDITIVA DE ORACIONES	DISFUNCIONAL		FUNCIONAL
	Ausencia de respuesta o nula comprensión auditiva.	Señala imagen incorrecta. Respuesta en > 10´	Señala la imagen correcta.
**Ítem 32**			
**Ítem 33**			
**Ítem 34**			
**Recuento Respuestas**			
**Etiqueta diagnóstica: Comprensión auditiva de oraciones disfuncional**	Si dos respuestas son disfuncioanles; una respuesta disfuncional si se trata del ítem 32		

COMPRENSIÓN AUDITIVA DE ÓRDENES VERBALES	DISFUNCIONAL		FUNCIONAL
	Ausencia de respuesta o nula comprensión auditiva.	La orden se cumple de manera incompleta o inadecuada. Respuesta en > 10˝	Ejecuta la orden de manera correcta.
**Ítem 35**			
**Ítem 36**			
**Ítem 37**			
**Recuento Respuestas**			
**Etiqueta diagnóstica: Comprensión auditiva de órdenes verbales disfuncional**	Si dos o más respuestas son disfuncioanles; una respuesta disfuncional si se trata del ítem 35.		

COMPRENSIÓN LECTORA DE PALABRAS	DISFUNCIONAL		FUNCIONAL
	Ausencia de respuesta o nula comprensión auditiva.	Señala una palabra incorrecta. Respuesta en > 15´´	Señala la respuesta correcta.
**Ítem 38**	☐	☐	☐
**Ítem 39**	☐	☐	☐
**Ítem 40**	☐	☐	☐
**Recuento Respuestas**			
**Etiqueta diagnóstica: Comprensión lectora de palabras disfuncional**	Si dos o más respuestas son disfuncionales		

COMPRENSIÓN LECTORA DE ORACIONES	DISFUNCIONAL		FUNCIONAL
**Ítem 41**	☐	☐	☐
**Ítem 42**	☐	☐	☐
**Ítem 43**	☐	☐	☐
**Recuento Respuestas**			
**Etiqueta diagnóstica: Comprensión lectora de oraciones disfuncional**	Si dos o más respuestas son disfuncionales		

Los símbolos pictográficos utilizados son propiedad del Gobierno de Aragón y han sido creados por Sergio Palao para ARASAAC (http://www.arasaac.org), que los distribuye bajo Licencia Creative Commons BY-NC-SA.

ARASAAC
